# Effect of nutritional counselling for using anthropometric indices among Indians

**DOI:** 10.6026/97320630018583

**Published:** 2022-06-30

**Authors:** Sanjay Chavan, Sanjana Challagalla, Shradha Salunkhe, Amodini Arora, Mayur Sharma, Sharad Agarkhedkar

**Affiliations:** 1 Department of Paediatrics, Dr. D.Y. Patil Medical College, Dr D.Y. Patil Vidyapeeth (DPU), Pimpri, Pune, Maharashtra, India - 411018

**Keywords:** malnutrition, nutritional counselling, diet

## Abstract

Malnutrition in children under 5 years is a major public health problem in developing countries. Malnutrition complex comprises of under-nutrition including multiple conditions like acute, chronic malnutrition, micronutrient deficiencies and nutrition related to obesity. Therefore, it is of interest to report data on the individualized nutritional counselling on nutritional status among mild to moderately malnourished children aged 2 - 5 years at one, three and six month follow up. Their dietary habits will help to determine the aetiology of mild/moderate malnutrition.150 Children attending outpatient department of the Department of Paediatrics of D Y Patil Medical College and admitted with mild/moderate malnutrition were included in the study after taking informed consent from their parents. The children were randomly allocated into 4 groups (group 1 - 4). The groups consisted of children where dedicated nutritional counselling was provided at 1 month, 1 and 3 month follow up and where-in no dedicated counselling was provided. A diet chart was provided with counselling. Group 4 followed up with routine care without any dietary intervention. Detailed dietary, socio economic history, clinical examination with anthropometry was done followed-up at 1, 3, 6 months from date of inclusion. Majority (57.3%) belonged to lower socio-economic class according to Kuppuswamy scale. The mean birth weight was 2.4 kg, age 34.5 months and age of weaning 7.6 months. Data shows that 70% children had mild malnutrition and 30% moderate malnutrition. At 6 month follow up amongst 105 children with mild malnutrition, 82 still had mild malnutrition, 4 normal, 19 had moderate malnutrition. Under-five childhood malnutrition is highly prevalent in poor socioeconomic strata of the society. Nutritional counselling provided by trained healthcare providers in existing settings are effective in improving nutritional status, daily calorie/protein intake, prevention of malnutrition. Prevention/treatment of co-existing illness bears equal importance.

## Background:

Malnutrition in fewer than 5 children is a major public health problem in developing countries. Malnutrition has economic, health, and social consequences for future generations. Malnutrition complex comprises of multiple conditions like chronic malnutrition, micronutrient deficiencies and nutrition related to obesity [1]. Chronic malnutrition is due to deficient intake/mal absorption of essential nutrients for a prolonged period. Stunting (short stature for age) - most commonly used indicator of chronic malnutrition, is related to developmental impairments and reduced financial potential later in life [2,3]. Anthropometric parameters such as weight, height, BMI for age and wasting (weight for height irrespective of age) are utilized for assessing nutritional status among children [4]. Malnutrition leads to an early physical growth failure, diminished immunity, delayed motor, cognitive, behavioural development, increased morbidity and mortality [5]. Since visible signs are not always present, micronutrient deficiencies are known as hidden hunger. Effect of chronic malnutrition is marked during initial years of life when rapid growth & development occur. Under nutrition remains an important public health problem in India, statistically proven. In India the prevalence of underweight children is among the highest in the world [6] and the burden of under-nutrition amongst <5 year children have not improved much despite intervention programs. It is reported that under-weight amongst <5 year was ranging from 39% to 75%, stunting from 15.4% to 74% and wasting from 10.6% to 42.3% in various parts of the country [6]. Amongst the developing nations, nutritional status of children was affected by place of residence, household wealth, maternal education, birth weight, age of child, drinking water source , number of <5 year children, diarrheal disease awareness and acute respiratory tract infection control [7]. Socioeconomic factors like illiteracy, poverty, inadequate quality of food items, large family and poor sanitary environment are the key factors [8]. According to NFHS 4 data, < 5 year mortality rate in India was 50 per 1000 live births [9]. In preschool children, the risk of infection was more constantly related with BMI (body mass index) for age and wasting indicating current energy insufficiency as compared to weight for age and height for age [10]. Majority of under-nutrition cases belonging to mild malnutrition category and distribution of double ration for them might be an important step in dealing with this problem [11]. Therefore, it is of interest to evaluate the dietary habits of mild to moderate malnourished children and effect of nutritional counselling at one, three and six months follow ups, the current study has been planned among 2 - 5 years old children.

## Materials and methods:

### Place of study:

Department of paediatrics at Dr D.Y. Patil Medical College, Hospital and Research centre, Pimpri, Pune.

### Duration of Study:

Period of 2 years, Ethics Committee clearance was obtained pre study.

### Type of study:

Randomized control and observational study.

### Inclusion criteria:

150 children with Mild or moderate malnutrition between 2 to 5 years admitted or attending, outpatient department in our hospital were included.

### Exclusion criteria:

[1] Severe acute malnutrition with or without complications.

[2] Children with chromosomal and congenital anomalies.

[3] Children lost to follow up.

[4] Children with secondary cause of malnutrition.

[5] Patient not consenting for the study.

### Methodology:

Children attending OPD or admitted with mild/moderate malnutrition in our hospital have been included in study after informed consent. The children were randomly allocated as follows:

Group I: Dedicated nutritional counselling was done on the day of inclusion.

Group II: Dedicated nutritional counselling was done on the day of inclusion and at one month follow-up visit.

Group III: Dedicated nutritional counselling was done on day of inclusion, one month and three month follow up visit.

Group IV: No dedicated specific nutritional counselling done and treated as per routine care of our hospital. A diet chart was made for Group 1, 2 and 3. Group 4 patients were followed up with routine care without any dietary intervention. At the time of enrolment- Detailed history with emphasis on dietary, socioeconomic, clinical examination, anthropometry was done. All children included in study were followed up after 1, 3 and 6 months from date of inclusion (+/-7days). At the time of follow up- Clinical examination, dietary history, nutritional assessment, recorded. Records of any illness, admissions, OPD visit from the last follow up were taken.

### Uses:

Anthropometry:

[1] Weight- Weight of the child is useful for calculating the right dosage of drugs.

[2] Height- Measured using Harpenden Stadiometer. The child was made to stand against a wall with bare feet touching, the heel, calf, buttock, upper back and occiput touching the wall and child looking straight.

[3] Mid arm circumference- Measured with a measuring tape mid-way between the acromion process and olecranon process with arm hanging by the side of the body. More than 13.5 cm considered normal, less than 12.5 indicates malnutrition.

## Results:

The study includes 150 children either mildly or moderately malnourished at inclusion and was assessed at 6 months’ follow up for anthropometric indices, daily calorie, and protein intake (Tables 1 to 4 - see PDF). An almost equal proportion of male and female patients (51.3% and 48.7% respectively) seen.57.3% children belonged to the lower socio-economic class, 42.7% came from upper-lower socio-economic class according to the modified Kuppuswamy scale. Mean birth weight of the children in study was 2.4 kg ± 0.1 kg (range 2.4 to 2.7 kg), mean age was 34.5 months ± 7 months (range 24 to 51 months). Mean age at which weaning was started was 7.6 months ± 1 month (range 5 to 9 months). Majority of the children were fed a vegetarian diet (111/150, 74%) as against only 39/150 (26%) fed non-vegetarian diet. A significant association between poor socioeconomic statuses (as defined by modified Kuppuswamy scale), birth weight < 2.5 kg, predominantly vegetarian diet, and higher prevalence of moderate malnutrition was found. The odds of a child from lower SE class being moderately malnourished were higher (OR 2.6, 95% CI 1.3 – 4.8) as compared to one from upper lower SE class and higher odds of being fed a vegetarian diet leading to a protein deficit (OR 2.5, 95% (1.1 - 5.1). Similarly, a child had higher odds of being born as a low-birth-weight infant (birth weight < 2.5kg) if the child belonged to the lower SE class (OR 3.5, 95% CI 1.8 - 7). Sample divided into 4 groups based on timing of dedicated nutritional counselling. Amongst 150 children, groups I, II and III had 40 (26.7%) each, while group IV had 30 (20%). There are an almost equal proportion of girls/ boys in all 4 groups. Cross-tabulation using chi-squared test revealed the difference in the proportion in all the groups was insignificant (p=0.587).Amongst 150 children, 105 (70%) had mild and 45 (30%) had moderate malnutrition at inclusion. At 1 month 68.7% had mild malnutrition and 31.3% had moderate malnutrition. 3 months follow up- 99/150 (66%) children had mild malnutrition 48/150, (32%) had moderate malnutrition, 3 children (2%) had severe malnutrition. At 6 months, out of 105 children with mild malnutrition, 82 had mild malnutrition, 19 moderate malnutrition of which 5, 3, 2, 9 from the 1st to 4th group respectively. 4 children were normal of which 3 from 1st group, 1 from 2nd group. Among 45 children with moderate malnutrition, 2 became mildly malnourished, 39 remained moderately malnourished, 4 became severely malnourished of which 2 were from 2nd group, 1 from the 3rd, 4th group each. Using one-way ANOVA, mean daily calorie intake of children was compared between Visit 1 (at inclusion) and subsequent visits at 1st, 3rd and 6th month follow up, namely Visit 2, Visit 3 and Visit 4 respectively. In Group I, the mean daily calorie intake increased per visit, however was not statistically significant (p=0.082). Group II - A consistent increase in daily calorie intake per visit. However, it is statistically insignificant. (p=0.066). Group III Increase was persistently high, and highly statistically significant (p=0.003). Group IV Mean daily calorie intake was low even at inclusion, however increased per visit and was statistically significant (p=0.0001). However, the mean daily calorie intake achieved at the last follow up by this group was much lower than the mean daily calorie intake of all other groups at baseline itself. Using one-way ANOVA, mean values of daily protein intake compared between time of inclusion (Visit 1) and per follow up, wasn't significantly different amongst 4 groups. At subsequent visits it increased per group however statistically insignificant increase in all the 4 groups (p>0.005).Mean weight comparison between inclusion wasn't significantly different compared to subsequent follow ups irrespective of allocation to any study group (p>0.05). However, the mean weight in Group IV remained lesser in comparison to three groups. There was an increase in the mean height of all the children per follow up visit but statistically insignificant (p>0.05).The mid-arm circumference of all the children in the study was comparable to begin with at the time of inclusion. At subsequent follow ups no change was seen in children belonging to groups I and IV, slight increase in groups II and III was noticed, but statistically insignificant (p>0.05).

## Discussion:

Malnutrition is defined as lack of proper nutrition caused by unavailability of food, inadequate staple dietary intake required for appropriate growth/nourishment of children. Malnutrition is a major public health problem affecting more than 30% under 5 children-developing countries, 50.6 million are undernourished. Children belonging to the severely malnourished category with a illness have led to a case-fatality rate exceeding 20% [12,13]. The prevalence of anaemia is significant in underdeveloped nations, and it is primarily due to iron deficiency and other factors. Low-income people are more likely to suffer from dietary inadequacies [14]. The study focused on nutritional status of 2-5-year children, impact of nutritional counseling at intervals as compared to children where no nutritional counseling was provided. The 150 children who were either mildly/moderately malnourished were included and assessed at 6 months for anthropometric indices, daily calorie, protein intake. The Gender distribution was equal, 51.3% male, 48.7% females. Similar observations were seen by Manjunath et al. (51.5% male, 47.5% female), Purohit et al. in this study the mean age was 34.5 months in a sample of 150. Purohit et al. in a Maharashtrian study reported a similar mean age at inclusion of 36 months [15]. Majority of the children belonged to lower socioeconomic class as per modified Kuppuswamy scale. Socioeconomic inequality in malnutrition among children has been documented by various studies from India [16], South Africa [17], China [18] and Ghana [8]. A Vietnamese study, reported highest prevalence of adverse nutritional status in socio economically deprived mountainous, midland areas [19]. A Nigerian study reported that childhood malnutrition was associated with poor socioeconomic status, defined by poor maternal income and their dwelling type was in a 1-bedroom household [20]. A Tehran report showed significant association between poorer society sections and malnutrition in fewer than 5 children [4]. Our finding was similar to the study done by Fall et al. in 2021. They stated that undernutrition (stunting and low BMI) was more prevalent than overweight or obesity. It was most widespread in the least developed rural areas and in Mumbai's slums, where obesity was unusual [21]. Ahmad Isah et al. study, for understanding socioeconomic characteristics of families with undernourished children, reported that undernutrition is more prevalent in children of petty traders, farmers, protein intake was inadequate, livestock rarely owned/slaughtered [22]. The Need for implementation of nutritional intervention strategies to bring improvement in household food security, child’s food intake must be emphasized [23]. Problem in complementary feeding practices in developing countries is caretakers' lack of knowledge of food and its preparation beneficial for growth/development. An important intervention is providing animal source protein and giving nutritional counselling to caretakers [24]. Such interventional programs were successful in Brazil [26], Peru [28], China [27], and Bangladesh [25]. A randomized controlled trial [29] in India to determine impact of nutritional education/ counselling proved that counselling provided by health care workers through existing primary healthcare services was effective in improving anthropometry, reducing illness episodes and increasing daily calorie intake in all groups enrolled. Which was also observed in a Vietnamese study by Pachon [19] 105 children with mild malnutrition at inclusion, reduced to 82 while 4 entered the normal category. Their mean daily calorie, protein intake increased. At 6 months, in Group 1 no significant increase in the daily calorie intake found. However, in Group 2, 3 the caloric intake significantly increased. The dietary pattern in these children had changed, showing that repeated counselling led to dietary pattern change. The mean weight of the children increased over 6 months, however statistically insignificant. In the study, it was observed that MAC didn’t show any significant change over 6 months in any of the 4 groups.

## Conclusion:

Under-five childhood malnutrition is highly prevalent in poor socioeconomic strata of the society. Nutritional counselling provided by trained healthcare providers in existing settings is effective in improving nutritional status, daily calorie/protein intake, prevention of malnutrition. Furthermore, to enhance the nutritional condition of families, there is a need to provide coordinated nutrition education to mothers.
